# *Echinochloa* Chloroplast Genomes: Insights into the Evolution and Taxonomic Identification of Two Weedy Species

**DOI:** 10.1371/journal.pone.0113657

**Published:** 2014-11-26

**Authors:** Chu-Yu Ye, Zhangxiang Lin, Gengmi Li, Ying-Ying Wang, Jie Qiu, Fei Fu, Haiqiang Zhang, Li Chen, Sisi Ye, Weijie Song, Gulei Jin, Jinwen Zhu, Yongliang Lu, Longbiao Guo, Longjiang Fan

**Affiliations:** 1 Department of Agronomy, College of Agriculture and Biotechnology, Zhejiang University, Hangzhou, China; 2 State Key Laboratory of Rice Biology, China National Rice Research Institute, Chinese Academy of Agricultural Sciences, Hangzhou, China; 3 Guhe info Corp., Hangzhou, China; 4 Department of Plant Protection, College of Agriculture and Biotechnology, Zhejiang University, Hangzhou, China; Nanjing Agricultural University, China

## Abstract

The genus *Echinochloa* (Poaceae) includes numerous problematic weeds that cause the reduction of crop yield worldwide. To date, DNA sequence information is still limited in the genus *Echinochloa*. In this study, we completed the entire chloroplast genomes of two *Echinochloa* species (*Echinochloa oryzicola* and *Echinochloa crus-galli*) based on high-throughput sequencing data from their fresh green leaves. The two *Echinochloa* chloroplast genomes are 139,891 and 139,800 base pairs in length, respectively, and contain 131 protein-coding genes, 79 indels and 466 substitutions helpful for discrimination of the two species. The divergence between the genus *Echinochloa* and *Panicum* occurred about 21.6 million years ago, whereas the divergence between *E. oryzicola* and *E. crus-galli* chloroplast genes occurred about 3.3 million years ago. The two reported *Echinochloa* chloroplast genome sequences contribute to better understanding of the diversification of this genus.

## Introduction

The genus *Echinochloa* (Poaceae) belongs to the subfamily Panicoideae [1], [2], [3]. This genus includes numerous problematic weeds that cause the reduction of crop yield worldwide. For example, *Echinochloa crus-galli* (L.) Beauv., which is distributed in paddy or non-paddy fields (such as upland crop areas), is considered as one of the most serious weeds [4], [5], [6]. Rice biomass is reduced by 75% and yield by 50% during cultivation with a ratio of 100 rice plants to 10 *E. crus-galli* plants [7]. Moon *et al.* (2010) showed that *E. crus-galli* plants significantly reduced number of rice tillers and resulted in significant reduction of rice yield during rice-*E. crus-galli* competition under transplanted rice cultivation [8]. *Echinochloa oryzicola* (Vasing.) Vasing. is common in paddy fields, and also reduces production of rice [9], [10]. The genus is comprised of approximately 50 species [11]. Taxonomic confusion remains in this genus, e.g., *E. oryzicola* is variously treated as *E. phyllopogon* (Stapf) Kossenko, *E. crus-galli* var. *oryzicola* (Vasing.) T. Koyama or a tetraploid *E. crus-galli* var. *oryzicola*. *E. oryzicola* has also been misidentified as *E. crus-galli* var. *formosensis* Ohwi [12], [13], [14]. To solve the difficulties of species identification and to understand the inter-specific genetic relationship of this genus, molecular techniques have been applied in several studies. Hilu (1994) assessed the proposed phylogeny and examined the genetic diversity in two domesticated species (*E. utilis* Ohwi & Yabuno and *E. frumentacea* Link.) and their wild counterpart using the random amplified polymorphic DNA markers [15]. Yasuda *et al.* (2002) discriminated *E. oryzicola* and *E. crus-galli* by the polymerase chain reaction-restriction fragment length polymorphism analysis [9]. An amplified fragment length polymorphism analysis on 80 accessions from Italian rice fields indicated two molecular groups [16]. Based on several non-coding region sequences (*trn*T-L, *trn*L-F intergenic spacers, and *trn*L intron) of chloroplast DNA, a phylogenetic tree grouped 30 accessions belonging to nine species of the genus *Echinochloa* into five [13]. Aoki and Yamaguchi (2008) further examined the genetic relationship between *E. crus-galli* and *E. oryzicola*, assuming that Eurasian *E. crus-galli* (hexaploid) arises from the hybridization between tetraploid *E. oryzicola* (paternal donor) and an unknown diploid species (maternal donor) [17]. Nevertheless, DNA sequence information that could provide effective information for taxonomy, species identification, and phylogenetics is still limited in the genus *Echinochloa*.

Compared with the nuclear genome, the chloroplast genome has distinct features, e.g., haploid, maternal inheritance, and high conservation in gene content and genome structure [18]. Chloroplast genomes of higher plants typically range in size from 120 to 180 kilobase pairs (kb) with conserved quadripartite structure that is composed of two copies of a large inverted repeat (IR) and two sections of unique DNA, i.e., large single-copy regions (LSC) and small single-copy regions (SSC) [18], [19]. Chloroplast DNA sequence data has been used in numerous studies for understanding of the phylogenetic relationships of plants at species, genera and tribal levels and population genetic analyses [20]. For example, 10 complete chloroplast genomes from seven orchid species provided insights into the phylogeny of the genus *Cymbidium* [18]. Twelve chloroplast genomes from wheat, barley and rye were used for the evolutionary analysis of the Triticeae tribe [21]. The origin of populations of *Arabidopsis thaliana* was investigated based on chloroplast DNA sequences of 77 accessions [22]. Complete chloroplast genomes of 12 native and 5 invasive individuals of *Jacobaea vulgaris* were used for population studies [23]. A set of 100 chloroplast DNA primer pairs was used to study population genetics in monocots [24]. Although chloroplast DNA sequence is useful for molecular systematic studies, the features of chloroplast genome hinder the overall utility of chloroplast DNA sequence in evolutionary analyses [25]. Chloroplast DNA reveals only a half of the parentage in plants of hybrid origin because it is uniparentally (primarily maternally) inherited and haploid [25]. The relatively slow evolutionary rate of chloroplast DNA often fails to provide significant phylogenetic information at low taxonomic levels [26]. Several genome features of weed species make it hard to obtain the whole nuclear genome sequences, such as unclear genetic background and high rate of heterozygosity. To date, chloroplast genome sequences of several weeds have been determined, such as waterhemp (*Amaranthus tuberculatus*) [27], *Coix lacryma-jobi* [28], *Jacobaea vulgaris* [23], common milkweed (*Asclepias syriaca*) [29], and crofton weed (*Ageratina adenophora*) [20]. To obtain chloroplast genome sequences, purification of the chloroplast or PCR amplification prior to sequencing is commonly involved in conventional approaches [30]. However, the relatively slow evolutionary rate or conservation of chloroplast DNA and advances in DNA sequencing technology provide a new opportunity to obtain the chloroplast genome based on whole-genome high-throughput sequencing data without purification of the chloroplast. Yang *et al.* (2010) reported on the complete chloroplast genome sequence of *Phoenix dactylifera* that was obtained from genomic DNA sequenced by GS FLX [31]. Three Lemnoideae chloroplast genomes were obtained through high-throughput DNA sequencing of genomic DNA using the SOLiD platform [32]. Tangphatsornruang *et al*. (2010) reported the use of 454 sequencing technology to obtain the chloroplast genome sequence of mungbean [30].

Species identification and clear understanding of genetic relationship of *Echinochloa* are very important to control effectively these weeds. However, morphology-based classification is difficult for this genus because of diverse taxonomic opinions. Meanwhile, molecular systematic research is constrained by the limited chloroplast DNA sequence information of the genus *Echinochloa*. To provide more DNA sequence information and insights into evolution of the genus *Echinochloa*, we employed the new approach to construct the complete chloroplast genome sequences of two *Echinochloa* species (i.e., *E. oryzicola* and *E. crus-galli*) in the current study. Furthermore, we investigated the phylogenetic divergence time within the *Echinochloa* genus and among several closely related genera.

## Materials and Methods

### Plant materials

Five (STB01-05) and three (BTS01-03) *Echinochloa* plant samples (matured spikelet) were collected from two different fields (paddy and upland crop fields, respectively) in September, 2011, from Yuhang County, Hangzhou, Zhejiang Province in China (E119°57′, N30°17′). No specific permissions were required for the collection sites, and the study did not involve endangered or protected species. Collected seeds were used to germinate in the next year after field collection. Five plants of each accession were grown in 5L plastic pots (20.5 cm in diameter and 20 cm in depth; one plant per pot), filled with a 3∶1∶1 mixture of soil: peat: sand in a greenhouse at 28°C/25°C (day/night) with a 16 h photoperiod. Seed weights of each *Echinochloa* accession were determined by random samples of 100 full matured spikelets from five plants in each of the three replicates, and were compared by *t*-test. Based on morphological observation, STB03 and BTS02, which had typical morphological features of *E. oryzicola* and *E. crus-galli*, respectively, were selected for further study.

### Chromosome number counting

Mitotic chromosome numbers were determined through conventional acetocarmine method [33]. After the roots from STB03 and BTS02 plants grown in the greenhouse were cleaned at 10 am, root tips were pretreated with a solution of 0.7 mmol/L cycloheximide for 4 h. Fixation with Carnoy’s fluid for at least 12 h and enzymolysis using mixed enzymes (cellulase and pectinase) at 37°C for 70 min were subsequently performed. After dyeing with acetocarmine, root tips were squashed on slide glass, and metaphase cells were observed using an Olympus (BH-2) microscope.

### Phylogenetic analysis

DNA was extracted from green leaves of STB03 and BTS02 using routine protocol (CTAB) [34]. The nucleotide sequences of *trn*T-L-F region (*trn*T-L intergenic spacer region, *trn*L genomic region, and *trn*L-F intergenic spacer region) in the chloroplast genome of STB03 (*E. oryzicola*) and BTS02 (*E. crus-galli*) were amplified by PCR with primers CATTACAAATGCGATGCTCT and ATTTGAACTGGTGACACGAG. The sequences were then aligned to those of 30 *Echinochloa* accessions [13] using MAFFT (http://mafft.cbrc.jp/alignment/server/) [35]. A phylogenetic tree for 32 accessions (OTUs) was constructed using NJ method with substitution model of maximum composite likelihood and 1,000 bootstraps in MEGA. ML method was also used for tree construction with substitution model of Tamura-Nei and 1,000 bootstraps (http://www.megasoftware.net/) [36].

### Genome sequencing

For chloroplast genome sequencing, total DNA was extracted from green leaves of STB03 and BTS02. DNA was fragmented into 300–400 bp pieces. Libraries with 230 bp insertion size for Illumina Hiseq2000 sequencing platform were constructed according to the manufacturer’s instructions (Illumina). Twenty-five and 50 Gb genome data were obtained for STB03 and BTS02, respectively. Raw data was removed adaptors and qualified with Fastx-toolkit with Q30 and above 50 bp.

### Genome assembly and annotation

Bowtie2 (http://bowtie-bio.sourceforge.net/index.shtml) [37] was used to map clean reads with the chloroplast genome of *Panicum virgatum* (Genbank accession number NC_015990, the closest species available that belongs to the same tribe as *Echinochloa*) as reference [38]. *De novo* assembly of the mapped reads was then performed using CLC software (http://www.clcbio.com) with default settings (word size of 20, bubble size of 50, auto-detect paired distances, create simple contig sequences). The gaps were closed by GapCloser (http://soap.genomics.org.cn/soapdenovo.html) [39] and PCR amplification. The joining of different scaffolds was further closed by PCR amplification. Genomic regions with variations between STB03 and the reference (*P. virgatum*) chloroplast genomes and those between STB03 and BTS02 were verified through PCR amplification. All PCR products were sequenced by the Sanger method [40]. Primers are listed in Table S1. We performed annotation of the *Echinochloa* chloroplast genomes using DOGMA (http://dogma.ccbb.utexas.edu/) [41]. The annotated files were used to draw gene maps using GenomeVx tool (http://wolfe.gen.tcd.ie/GenomeVx/) [42]. The complete chloroplast genome sequences of two *Echinochloa* species were deposited into GenBank with accession numbers KJ000048 (STB03) and KJ000047 (BTS02), respectively.

### Sequence divergence analysis

The complete chloroplast genomes of *P. virgatum*, *Sorghum bicolor*, *Triticum aestivum*, and *Oryza sativa* were obtained from NCBI through accession numbers NC_015990, NC_008602, NC_002762, and NC_008155, respectively. The sequence identity was plotted using mVISTA (http://genome.lbl.gov/vista/index.shtml) with the default settings (sliding window size 100 bp and minimum width of a conserved region 100 bp) [43].

### Estimation of divergence times

Six species (*O. sativa*, *S. bicolor*, *Zea mays*, *P. virgatum*, *E. crus-galli* and *E. oryzicola*) were involved in the analysis on divergence time estimation. Multiple sequence alignments were conducted for all chloroplast genome sequences with MAFFT [35]. To optimize the alignment for further tree construction, Gblocks was used for the removal of poorly aligned positions [44]. The minimum length of a block was set to 5, and the maximum number of contiguous non-conserved positions allowed is 8. Plastid divergence times were estimated using an uncorrelated relaxed clock in BEAST (http://beast.bio.ed.ac.uk/Main_Page) with *O. sativa* as an outgroup [45]. Hasegawa-Kishino-Yano (HKY) model [46] of evolution with gene-specific gamma-distributed rate heterogeneity among sites and gene-specific evolutionary rates was applied for Bayesian MCMC analysis. Monophyletic constraints were imposed for the nodes that were used to calibrate the evolutionary rates. We used a Yule speciation process, which specifies a constant rate of species divergence [47]. Normal priors were used for the BEP-PACCAD split time (mean: 50.0 Mya, stdev: 4.0) and the *Z. mays*-*S. bicolor* split time (mean: 13.0 Mya, stdev: 1.0) [48]. The MCMC chains in BEAST were run for 20,000,000 generation sampling every 2,000 steps. Thus, a tree file containing 10,000 trees was generated and 20% burn in was specified to use the value 2,000. Convergence between the runs and the amount of burn in were determined using Tracer 1.5, which was used to assess the effective sample size (ESS) and to check the consistency of the result (Figure S1 and Table S2). Coding sequences of single copy genes shared among the six species were also used to estimate divergence time with the same method.

## Results and Discussion

### Species identification of STB03 and BTS02

**Figure 1:**
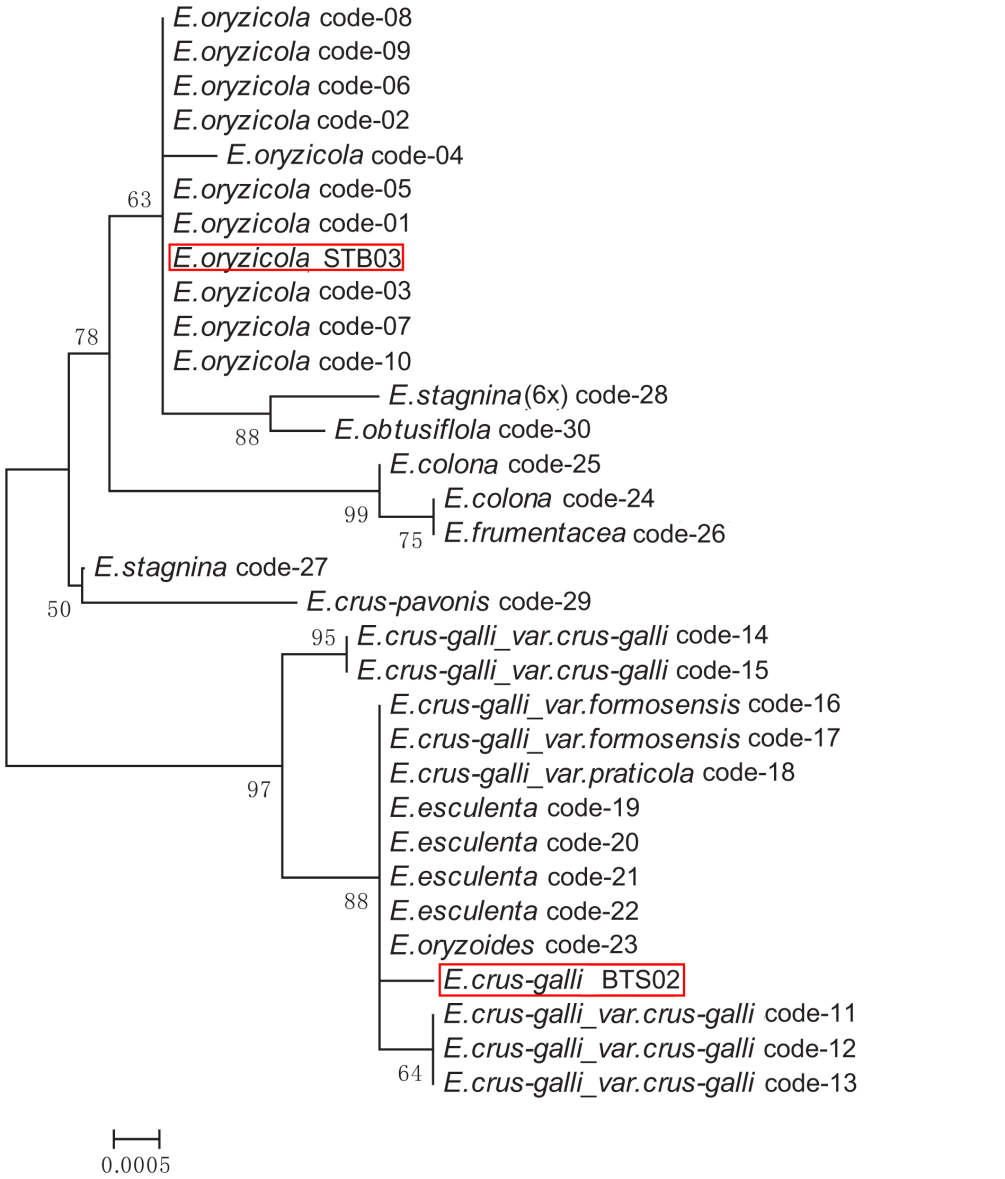
Phylogenetic relationships among STB03, BTS02, and 30 *Echinochloa* accessions based on the nucleotide sequences of *trn*T-L-F region of the chloroplast genome. See the study by Yamaguchi *et al.* (2005) for code numbers of the 30 accessions [12]. The tree was constructed using NJ method. Bootstrap values with less than 50 are not shown.

For our genome sequencing effort, species of our *Echinochloa* collection was first identified. In the five (STB01-05) and three (BTS01-03) *Echinochloa* plants collected in paddy and upland crop fields, respectively, STB03 had a compact plant type, bigger seeds (0.430 g per hundred spikelets), and high similarity with rice (similar plant architecture and leaf color at the seedling stage), which are typical morphological features of *E. oryzicola*. BTS02 had geniculate culms, smaller seeds (0.096 g per hundred spikelets, significantly smaller than that of STB03, *P*<0.001, *t*-test), and a loose plant type, which are typical morphological features of *E. crus-galli* [49], [50] (Figure S2). Chromosome number observation showed that STB03 has 36 chromosomes (2n = 4x), which is the same as *E. oryzicola* [10], [17]. By contrast, BTS02 has 54 chromosomes (2n = 6x), which is the same as *E. crus-galli* (Figure S3). To further identify the taxonomic species for STB03 and BTS02, the chloroplast regions of *trn*T-L-F were PCR amplified and sequenced. Their sequence alignment showed that STB03 has a 33 bp insertion compared with BTS02, demonstrating that STB03 has the same genotype as *E. oryzicola* and BTS02 as *E. crus-galli*. Taken together, we determined STB03 as *E. oryzicola* and BTS02 as *E. crus-galli*, and selected them for our further effort of genome sequencing. We also reconstructed ML and NJ phylogenetic trees using the sequences of *trn*T-L-F from the 30 accessions used in the study by Yamaguchi *et al.* (2005) [13] and our two species. Both trees (Figure 1 and Figure S4), with a same topology, showed that STB03 is grouped with *E. oryzicola* and BTS02 with *E. crus-galli*.

### Genome assembly

Three scaffolds were generated after collecting *E. oryzicola* (STB03) chloroplast-related reads and *de novo* assembly of these reads. Four gaps (each with size of <20 bp) within the three scaffolds and two inter-scaffold gaps (PCR production size of 947 and 931 bp, respectively) were closed by PCR amplification coupled with Sanger sequencing. After alignment of the STB03 assembly with the reference (*P. virgatum* chloroplast genome), the STB03 genome structure (IR, LSC and SSC) could be determined, showing that only one IR (IRa) was successfully assembled. PCR primers were then designed for the joining of IRb-LSC and IRb-SSC and the validation of two indels between STB03 and the reference. Finally, all clean reads generated by our high-throughput sequencing from STB03 were mapped back to the STB03 assembly. No SNPs were found, suggesting no variations between the two IR regions and also a high quality of our assembly. For the assembly of *E. crus-galli* (BTS02), the chloroplast genome of *E. oryzicola* (STB03) was used as the reference and four pairs of primers were designed for gap closure and joining regions of IR-LSC and IR-SSC. Meanwhile, PCR amplification followed by Sanger sequencing was used to validate eight regions with variations between *E. oryzicola* (STB03) and *E. crus-galli* (BTS02). Finally, we obtained complete chloroplast genomes of *E. oryzicola* and *E. crus-galli* with length of 139,891 and 139,800 bp, respectively.

The approach by whole-genome high-throughput sequencing without purification of the chloroplast DNA provides a new way to obtain the chloroplast genome and has been successfully used in several studies [28], [29], [30]. One obvious advantage for this method is that the purification of chloroplast DNA is not required prior to sequencing. Meanwhile, the chloroplast genome would be obtained from total DNA with even low coverage sequencing because of high copy number of chloroplast DNA. However, chloroplast genomes of one or several evolutionarily close species are necessary for the assembly of target genome. Numerous reads from nuclei and mitochondrion will affect the assembly when a chloroplast genome of evolutionarily close reference species is lacking. Wang and Messing (2011) compared the assembly from total reads with and without filtering by reference genome showing that masking non-chloroplast reads with a related genome sequence is critical for chloroplast genome assembly [32].

### Genome annotation

**Figure 2:**
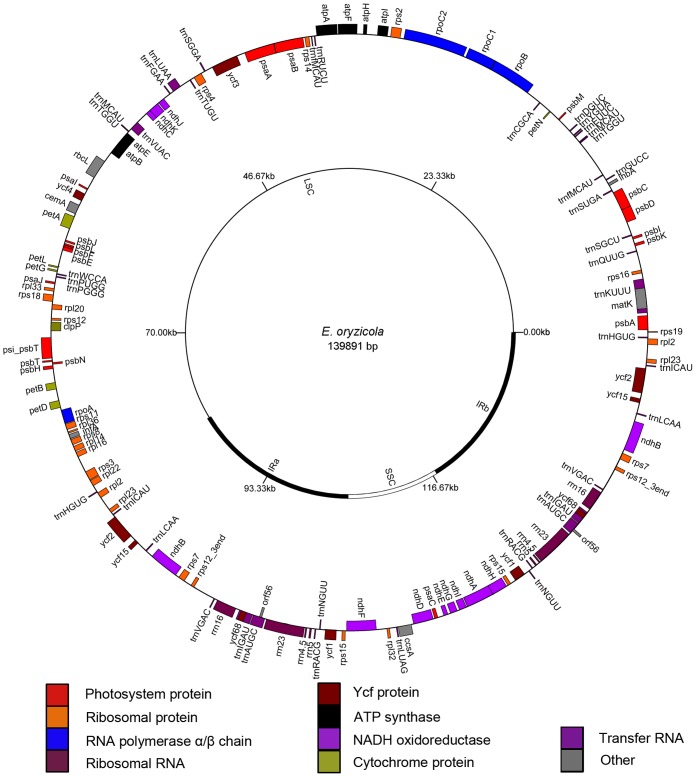
The *E. oryzicola* chloroplast genome structure and annotation. Outer circle: The genes shown outside of the circle are transcribed clockwise, whereas those inside are transcribed counterclockwise; Inner circle: the genomic structure with two inverted repeats (IR) and two single copy regions (LSC and SSC). Genes belonging to different functional groups are color coded.

Both *E. oryzicola* and *E. crus-galli* chloroplast genomes displayed typical quadripartite structure consisting of a pair of IRs (22,289 and 22,618 bp, respectively) separated by LSC (82,108 and 82,047 bp, respectively) and SSC (13,205 and 12,517 bp, respectively) regions (Figure 2 and Figure S5). The *Echinochloa* chloroplast genomes are AT-rich (61.37% in *E. oryzicola* and 61.38% in *E. crus-galli*), which is generally similar to other chloroplast genomes [31], [51]. Both chloroplast genomes encode 131 predicted genes, among which 112 are unique in the LSC/SSC regions and 19 are duplicated in the IR regions. The 112 unique genes include 33 transfer RNAs, 4 ribosomal RNAs, and 75 protein-coding genes in both chloroplast genomes. Genes with one intron include *atpF*, *ndhB*, *ndhA*, *trnK-*UUU, *trnT-*GGU, *trnL-*UAA, *trnV-*UAC, *trnI-*GAU, and *trnA-*UGC, whereas *ycf3* harbors two introns. Some genes should have become pseudogenes because of the early stop codons identified in their coding sequences, such as *rps19*, *rps16*, *ycf2*, *ycf15*, *orf56* and *ycf1*. The similar mutations have been observed in the chloroplast genomes of other angiosperm species [18], [28]. We did not find any genes with non-canonical start codons, which have been detected in some other species [18], [28]. No gene gain events were observed in the *Echinochloa* chloroplast genomes.

### Genome comparison

The two *Echinochloa* chloroplast genomes were conserved with 99.5% sequence identity and contained similar genes. However, a total of 79 indels (Table S3) and 466 substitutions between the two genomes were still found. The identification of the *Echinochloa* species has been difficult based on morphological feature. Different taxonomic systems on the genus have been proposed by several authors [16]. Although DNA-based taxonomy has limitations, the method remains an effective and universal tool in species identification [52]. Molecular techniques have been used to identify species and to investigate inter- and intra- specific genetic relationships of the genus [9], [13], [15], [16]. As compared with previous studies, the two complete chloroplast genome sequences and the genomic variations (79 indels and 466 substitutions) between them would provide more valid information for studies on genetic relationship of this genus.

**Figure 3:**
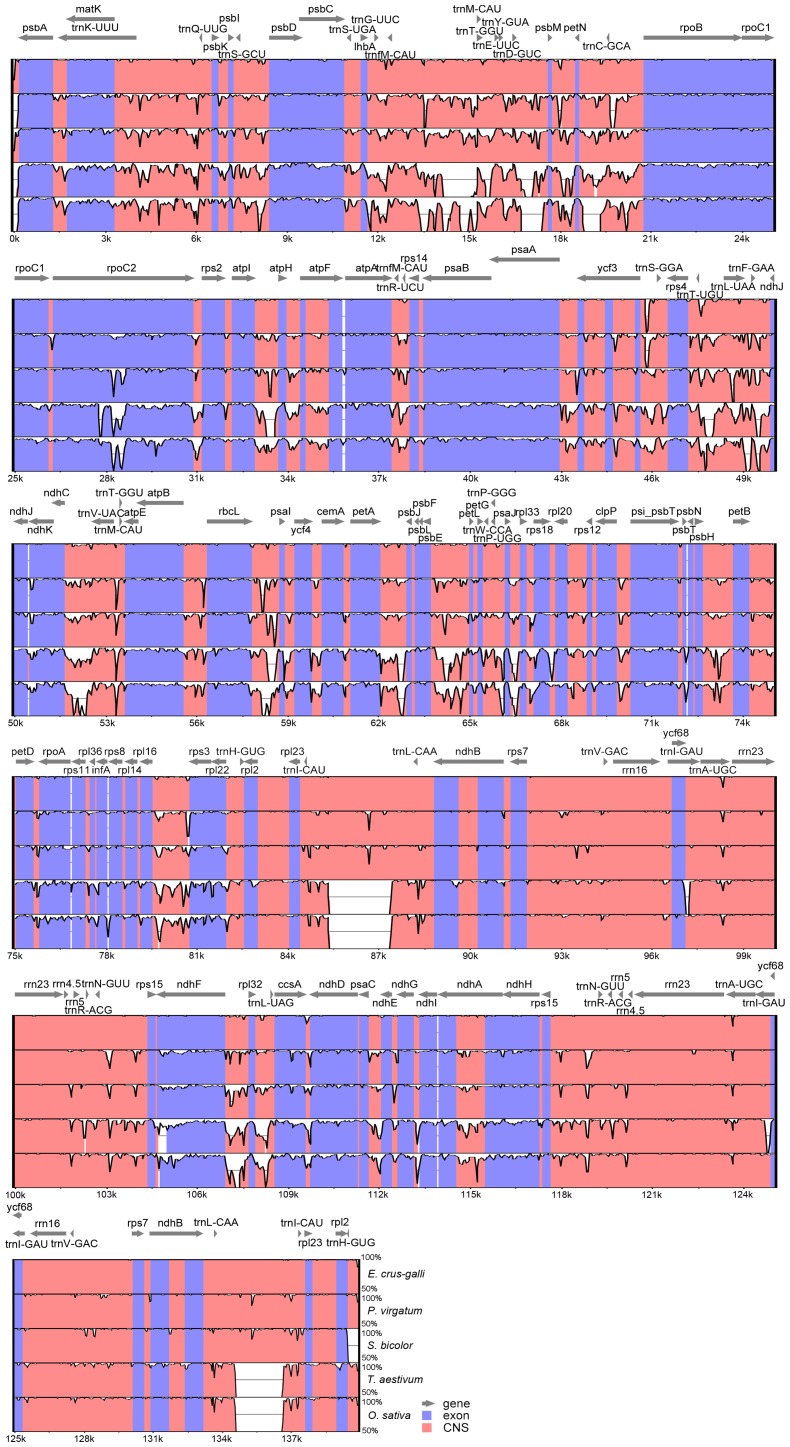
Visualization of alignments of chloroplast genome sequences. The sequence identity was plotted with the *E.oryzicola* chloroplast genome as the reference. Sequence identity with 50%–100% is shown. Exonic regions and conserved non-coding sequences (CNS) are colored in blue and red, respectively.

To further reveal the chloroplast genome divergence of *Echinochloa* and other Poaceae members (*P. virgatum*, *S. bicolor*, *T. aestivum* and *O. sativa*), sequence identity was plotted using mVISTA [43], with *E. oryzicola* as the reference (Figure 3). Results showed that the *Echinochloa* chloroplast genomes share high sequence identity with those of *P. virgatum* and *S. bicolor*, and relatively lower identity with those of *T. aestivum* and *O. sativa*. Particularly, several large genomic variations among them were observed. These results are generally consistent with the phylogeny of the grass family [1], [53].

Compared to other Poaceae members and *Arabidopsis* (Table S4), seven genes were lost or became pseudogenes in the *Echinochloa* chloroplast genomes. Among the seven genes, *accD* encoding one subunit of acetyl-CoA carboxylase and two genes (*ycf1* and *ycf2*) encoding two large open reading frames were frequently lost or became pseudogenes in Poaceae chloroplast genomes [18]. The ribosomal gene *rps16* also became a pseudogene in *O. sativa*, while *rps19* was only found as a pseudogene in the *Echinochloa* chloroplast genomes. Additionally, *ycf15* and *orf56* were lost or became pseudogenes in any of the species examined.

### Genome divergence time

**Figure 4:**
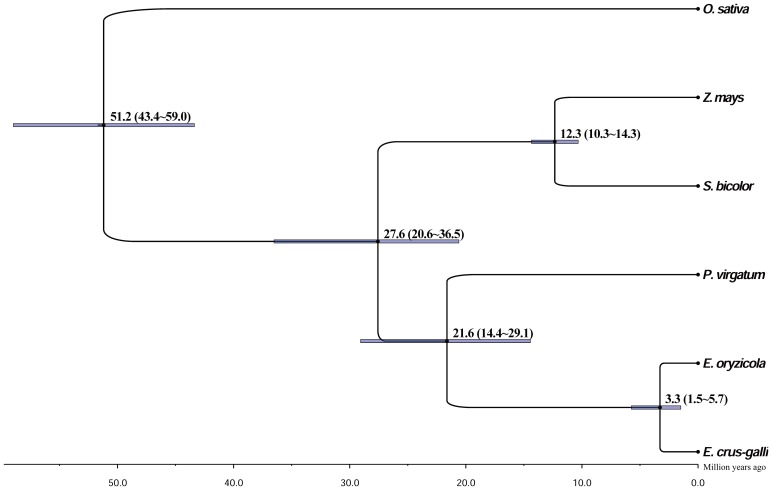
Divergence time of the genus *Echinochloa*. Divergence time was estimated using BEAST based on the complete chloroplast genomes of six species (*E. oryzicola*, *E. crus-galli*, *P. virgatum*, *S. bicolor*, *Z. mays*, and *O. sativa*). The numbers showed at nodes indicate divergence time.

To estimate the divergence time of *Echinochloa* species, chloroplast genomes from six members of the grass family (*E. oryzicola*, *E. crus-galli*, *P. virgatum*, *S. bicolor*, *Z. mays*, and *O. sativa*) were used. *O. sativa* was selected as an outgroup and *T. aestivum*, the member of the subfamily Pooideae, was excluded in this analysis. The constructed tree based on the whole complete chloroplast genomes shows that the *Echinochloa* species were first grouped with *P. virgatum* and then *S. bicolor* and *Z. mays* (Figure 4). According to the estimation, we propose that the genus *Echinochloa* branched off from the genus *Panicum* around 21.6 million years ago (Mya), and the divergence date between *E. oryzicola* and *E. crus-galli* was around 3.3 Mya. Meanwhile, we estimated the divergence times based on coding sequences of single copy genes, which are slightly younger than those estimated by the whole chloroplast genomes (Figure S6). It may be because coding sequences are relatively conserved.

The subfamily Panicoideae (Poaceae) is comprised of two major tribes, i.e., Paniceae that includes *P. virgatum* and *Setaria italica*, and Andropogoneae that includes *S. bicolor* and *Z. mays* [53]. Limited DNA information was used to study the evolutionary position of the genus *Echinochloa*. So far, studies on the molecular evolution of *Echinochloa* were based on sequences of selected genes or partial regions [13], [15], [16], [17]. However, it was possible for the first time to perform the whole chloroplast genome phylogenetic analysis on the evolution of this genus with our two finished chloroplast genomes. The phylogenetic tree clearly supports the previous result that *Echinochloa* species are close to *P. virgatum*, which belongs to the tribe Paniceae. Determining divergence time is helpful in interpreting patterns of speciation, estimating rates of genetic and morphological change, and understanding biogeographic history [54]. It is believed that *E. crus-galli* was derived from a hybrid between *E. oryzicola* (paternal donor) and an unknown diploid species (maternal donor) [10], [13], [17]. Chloroplast sequences only reflect a history of maternal factors. Thus, the 3.3 Mya divergent time estimated by chloroplast genome sequences should be close to the split point of *E. oryzicola* and the unknown diploid species. To determine the speciation time of *E. crus-galli*, i.e., the hybridization event of *E. oryzicola* and the unknown diploid maternal parent of *E. crus-galli*, nuclear DNA sequences are necessary.

In summary, we obtained the entire chloroplast genomes of two *Echinochloa* species, providing more DNA sequence information for genetic relationship and population studies on this genus. Furthermore, the phylogenetic divergence time estimated based on the chloroplast genome sequences could be useful to better understand the evolution of the genus *Echinochloa*.

## Supporting Information

Figure S1 **The first 20% (A, whole chloroplast genome sequences) and 10% (B, coding sequences of single copy genes) MCMC samples have been discarded as burn in.**
(PPT)

Figure S2 **The images of STB03and BTS02 show that the former has some typical morphological traits of** ***E. oryzicola*** **, such as a compact plant type, bigger seeds, and high similarity with rice, whereas the latter has traits of** ***E. crus-galli*** **, such as geniculate culms, smaller seeds, and a loose type.**
(PPT)

Figure S3 **Chromosome numbers show that STB03 (2n = 4x = 36) is tetraploid (A) and BTS02 (2n = 6x = 54) is hexaploid (B).** The numbers were determined by the conventional acetocarmine method.
(PPT)

Figure S4 **Phylogenetic relationships among STB03, BTS02, and 30** ***Echinochloa*** **accessions based on the nucleotide sequences of the** ***trn*** **T-L-F region of the chloroplast genome.** The tree was constructed using the ML method. Bootstrap values with less than 50 are not shown.
(PPT)

Figure S5 **The** ***E. crus-galli*** **chloroplast genome structure and annotation.** Outer circle: The genes shown outside of the circle are transcribed clockwise, whereas those inside are transcribed counterclockwise; Inner circle: the genomic structure with two inverted repeats (IR) and two single copy regions (LSC and SSC). Genes belonging to different functional groups are color coded.
(PPT)

Figure S6 **Divergence time of the genus** ***Echinochloa*** **.** Divergence time was estimated using BEAST based on coding sequences of single copy genes shared among the six species (*E. oryzicola*, *E. crus-galli*, *P. virgatum*, *S. bicolor*, *Z. mays*, and *O. sativa*). The numbers showed at nodes indicate divergence time.
(PPT)

Table S1 **Primers used in the assembly of two** ***Echinochloa*** **chloroplast genomes.**
(XLS)

Table S2 **The performed MCMC runs, burn in and the effective sample size (ESS).**
(XLS)

Table S3 **Indels found in the** ***Echinochloa*** **chloroplast genomes.**
(XLS)

Table S4 **Number of protein-coding genes in the seven chloroplast genomes (** ***E. oryzicola*** **,** ***E. crus-galli*** **,** ***P. virgatum*** **,** ***S. bicolor*** **,** ***T. aestivum*** **,** ***O. sativa*** **, and** ***A. thaliana*** **).**
(XLS)

## References

[1] Giussani LM, Cota-Sanchez JH, Zuloaga FO, Kellogg EA (2001) A molecular phylogeny of the grass subfamily Panicoideae (Poaceae) shows multiple origins of C4 photosynthesis. Am J Bot. 88:1993–2012. 21669633

[2] Clayton WD, Renvoize SA (1986) Genera Graminum. Grasses of the World. Kew Bulletin, Additional Series. 13:1–389.

[3] Tateoka T (1957) Miscellaneous papers on the phylogeny of Poaceae (10). Proposition of a new phylogenetic system of Poaceae. J Jap Bot. 32:275–287.

[4] Chauhan B, Johnson D (2011) Ecological studies on *Echinochloa crus-galli* and the implications for weed management in direct-seeded rice. Crop Protection. 30:1385–1391.

[5] Holm LG, Plucknett DL, Pancho JV, Herberger JP (1977) The World’s Worst Weeds: Distribution and Biology. University of Hawaii Press, Honolulu, Hawaii, USA.

[6] Rao AN, Johnson DE, Sivaprasad B, Ladha JK, Mortimer AM (2007) Weed management in direct-seeded rice. Adv Agron. 93:153–255.

[7] Graf B, Hill J (1992) Modelling the competition for light and nitrogen between rice and *Echinochloa crus-galli* . Agr Syst. 40:345–359.

[8] Moon BC, Cho SH, Kwon OD, Lee SG, Lee BW, et al (2010) Modelling rice competition with *Echinochloa crus-galli* and *Eleocharis kuroguwai* in transplanted rice cultivation. J Crop Sci Biotech. 13:121–126.

[9] Yasuda K, Yano A, Nakayama Y, Yamaguchi H (2002) Molecular identification of *Echinochloa oryzicola* Vasing. and *E. crus-galli* (L.) Beauv. using a polymerase chain reaction–restriction fragment length polymorphism technique. . Weed Biol Manag. 2:11–17.

[10] Yabuno T (1966) Biosystematic study of the genus *Echinochloa* . Jap J Bot. 19:277–323.

[11] Michael P (2003) *Echinochloa* P. Beauv. In: Flora of North America, North Mexico, Vol. 25 (ed. by Barkworth MEapels KMCLong S&Piep MB)Oxford University Press, Oxford 390–403.

[12] Aoki D, Yamaguchi H (2009) *Oryza* sh4 gene homologue represents homoeologous genomic copies in polyploid *Echinochloa* . Weed Biol Manag. 9:225–233.

[13] Yamaguchi H, Utano A, Yasuda K, Yano A, Soejima A (2005) A molecular phylogeny of wild and cultivated *Echinochloa* in East Asia inferred from non-coding region sequences of trnT-L-F. Weed Biol Manag. 5:210–218.

[14] Yabuno T (1981) Cytological relationship between *Echinochloa oryzicola* Vasing. and the french strain of *E. phyllopogon* Stapf subsp. *oryzicola* (Vasing.) Koss. Cytologia. 46:393–396.

[15] Hilu K (1994) Evidence from RAPD markers in the evolution of *Echinochloa* millets (Poaceae). Plant Syst Evol. 189:247–257.

[16] Tabacchi M, Mantegazza R, Spada A, Ferrero A (2006) Morphological traits and molecular markers for classification of *Echinochloa* species from Italian rice fields. Weed Sci. 54:1086–1093.

[17] Aoki D, Yamaguchi H (2008) Genetic relationship between *Echinochloa crus-galli* and *Echinochloa oryzicola* accessions inferred from internal transcribed spacer and chloroplast DNA sequences. Weed Biol Manag. 8:233–242.

[18] Yang JB, Tang M, Li HT, Zhang ZR, Li DZ (2013) Complete chloroplast genome of the genus *Cymbidium*: lights into the species identification, phylogenetic implications and population genetic analyses. BMC Evol Biol. 13:84. 23597078 10.1186/1471-2148-13-84 PMC3644226

[19] Shaw J, Lickey EB, Schilling EE, Small RL (2007) Comparison of whole chloroplast genome sequences to choose noncoding regions for phylogenetic studies in angiosperms: the tortoise and the hare III. Am J Bot. 94:275–288. 21636401 10.3732/ajb.94.3.275

[20] Nie X, Lv S, Zhang Y, Du X, Wang L, et al (2012) Complete chloroplast genome sequence of a major invasive species, crofton weed (*Ageratina adenophora*). PLoS One. 7:e36869. 22606302 10.1371/journal.pone.0036869 PMC3350484

[21] Middleton CP, Senerchia N, Stein N, Akhunov ED, Keller B, et al (2014) Sequencing of chloroplast genomes from wheat, barley, rye and their relatives provides a detailed insight into the evolution of the Triticeae tribe. PLoS One. 9:e85761. 24614886 10.1371/journal.pone.0085761 PMC3948623

[22] Yin P, Kang J, He F, Qu LJ, Gu H (2010) The origin of populations of *Arabidopsis thaliana* in China, based on the chloroplast DNA sequences. BMC Plant Biol. 10:22. 20141622 10.1186/1471-2229-10-22 PMC2827422

[23] Doorduin L, Gravendeel B, Lammers Y, Ariyurek Y, Chin AWT, et al (2011) The complete chloroplast genome of 17 individuals of pest species *Jacobaea vulgaris*: SNPs, microsatellites and barcoding markers for population and phylogenetic studies. DNA Res. 18:93–105. 21444340 10.1093/dnares/dsr002 PMC3077038

[24] Scarcelli N, Barnaud A, Eiserhardt W, Treier UA, Seveno M, et al (2011) A set of 100 chloroplast DNA primer pairs to study population genetics and phylogeny in monocotyledons. PLoS One. 6:e19954. 21637837 10.1371/journal.pone.0019954 PMC3102674

[25] Small RL, Cronn RC, Wendel JF (2004) LAS Johnson Review No. 2. Use of nuclear genes for phylogeny reconstruction in plants. Aust Syst Bot. 17:145–170.

[26] Small RL, Ryburn JA, Wendel JF (1999) Low levels of nucleotide diversity at homoeologous Adh loci in allotetraploid cotton (*Gossypium* L.). Mol Biol Evol. 16:491–501. 10331275 10.1093/oxfordjournals.molbev.a026131

[27] Lee RM, Thimmapuram J, Thinglum KA, Gong G, Hernandez AG, et al (2009) Sampling the waterhemp (*Amaranthus tuberculatus*) genome using Pyrosequencing technology. Weed Sci. 57:463–469.

[28] Leseberg CH, Duvall MR (2009) The complete chloroplast genome of *Coix lacryma-jobi* and a comparative molecular evolutionary analysis of plastomes in cereals. J Mol Evol. 69:311–318. 19777151 10.1007/s00239-009-9275-9

[29] Straub SC, Fishbein M, Livshultz T, Foster Z, Parks M, et al (2011) Building a model: developing genomic resources for common milkweed (*Asclepias syriaca*) with low coverage genome sequencing. BMC Genomics. 12:211. 21542930 10.1186/1471-2164-12-211 PMC3116503

[30] Tangphatsornruang S, Sangsrakru D, Chanprasert J, Uthaipaisanwong P, Yoocha T, et al (2010) The chloroplast genome sequence of mungbean (*Vigna radiata*) determined by high-throughput pyrosequencing: structural organization and phylogenetic relationships. DNA Res. 17:11–22. 20007682 10.1093/dnares/dsp025 PMC2818187

[31] Yang M, Zhang X, Liu G, Yin Y, Chen K, et al (2010) The complete chloroplast genome sequence of date palm (*Phoenix dactylifera* L.). PLoS One. 5:e12762. 20856810 10.1371/journal.pone.0012762 PMC2939885

[32] Wang W, Messing J (2011) High-throughput sequencing of three Lemnoideae (duckweeds) chloroplast genomes from total DNA. PLoS One. 6:e24670. 21931804 10.1371/journal.pone.0024670 PMC3170387

[33] Tsuchiya T (1971) An improved acetocarmine squash method, with special reference to the modified Rattenbury’s method of making a preparation permanent. Barley Genet Newsl. 1:71–72.

[34] Murray MG, Thompson WF (1980) Rapid isolation of high molecular weight plant DNA. Nucleic Acids Res. 8:4321–4325. 7433111 10.1093/nar/8.19.4321 PMC324241

[35] Katoh K, Standley DM (2013) MAFFT multiple sequence alignment software version 7: improvements in performance and usability. Mol Biol Evol. 30:772–780. 23329690 10.1093/molbev/mst010 PMC3603318

[36] Tamura K, Stecher G, Peterson D, Filipski A, Kumar S (2013) MEGA6: Molecular evolutionary genetics analysis version 6.0. Mol Biol Evol. 30:2725–2729. 24132122 10.1093/molbev/mst197 PMC3840312

[37] Langmead B, Salzberg SL (2012) Fast gapped-read alignment with Bowtie 2. Nat Methods. 9:357–359. 22388286 10.1038/nmeth.1923 PMC3322381

[38] Young HA, Lanzatella CL, Sarath G, Tobias CM (2011) Chloroplast genome variation in upland and lowland switchgrass. PLoS One. 6:e23980. 21887356 10.1371/journal.pone.0023980 PMC3161095

[39] Li R, Zhu H, Ruan J, Qian W, Fang X, et al (2010) *De novo* assembly of human genomes with massively parallel short read sequencing. Genome Res. 20:265–272. 20019144 10.1101/gr.097261.109 PMC2813482

[40] Sanger F, Nicklen S, Coulson AR (1977) DNA sequencing with chain-terminating inhibitors. Proc Natl Acad Sci U S A 74:5463–5467. 271968 10.1073/pnas.74.12.5463 PMC431765

[41] Wyman SK, Jansen RK, Boore JL (2004) Automatic annotation of organellar genomes with DOGMA. Bioinformatics. 20:3252–3255. 15180927 10.1093/bioinformatics/bth352

[42] Conant GC, Wolfe KH (2008) GenomeVx: simple web-based creation of editable circular chromosome maps. Bioinformatics. 24:861–862. 18227121 10.1093/bioinformatics/btm598

[43] Mayor C, Brudno M, Schwartz JR, Poliakov A, Rubin EM, et al (2000) VISTA: visualizing global DNA sequence alignments of arbitrary length. Bioinformatics. 16:1046–1047. 11159318 10.1093/bioinformatics/16.11.1046

[44] Castresana J (2000) Selection of conserved blocks from multiple alignments for their use in phylogenetic analysis. Mol Biol Evol. 17:540–552. 10742046 10.1093/oxfordjournals.molbev.a026334

[45] Drummond AJ, Suchard MA, Xie D, Rambaut A (2012) Bayesian phylogenetics with BEAUti and the BEAST 1.7. Mol Biol Evol. 29:1969–1973. 22367748 10.1093/molbev/mss075 PMC3408070

[46] Hasegawa M, Kishino H, Yano T (1985) Dating of the human-ape splitting by a molecular clock of mitochondrial DNA. J Mol Evol. 22:160–174. 3934395 10.1007/BF02101694

[47] Yule GU (1924) A mathematical theory of evolution: based on the conclusions of Dr. J.C. Willis. Philos Trans R Soc Ser B. 213:21–87.

[48] Zhang G, Liu X, Quan Z, Cheng S, Xu X, et al (2012) Genome sequence of foxtail millet (*Setaria italica*) provides insights into grass evolution and biofuel potential. Nat Biotechnol. 30:549–554. 22580950 10.1038/nbt.2195

[49] Liebman M (2001) Ecological management of agricultural weeds. Cambridge University Press. 217 p.

[50] Clayton WD, Vorontsova MS, Harman KT, Williamson H (2006) GrassBase - The online world grass flora. Available: http://www.kew.org/data/grasses-db.html. Accessed 08 November 2006.

[51] Raubeson LA, Peery R, Chumley TW, Dziubek C, Fourcade HM, et al (2007) Comparative chloroplast genomics: analyses including new sequences from the angiosperms *Nuphar advena* and *Ranunculus macranthus* . BMC Genomics. 8:174. 17573971 10.1186/1471-2164-8-174 PMC1925096

[52] Tautz D, Arctander P, Minelli A, Thomas R, Vogler A (2003) A plea for DNA taxonomy. Trends Ecol Evol. 18:70–74.

[53] Aliscioni SS, Giussani LM, Zuloaga FO, Kellogg EA (2003) A molecular phylogeny of *Panicum* (Poaceae: Paniceae): tests of monophyly and phylogenetic placement within the Panicoideae. Am J Bot. 90:796–821. 21659176 10.3732/ajb.90.5.796

[54] Zou XH, Yang Z, Doyle JJ, Ge S (2013) Multilocus estimation of divergence times and ancestral effective population sizes of *Oryza* species and implications for the rapid diversification of the genus. New Phytol. 198:1155–1164. 23574344 10.1111/nph.12230

