# Supplementary material for: Echinochloa Chloroplast Genomes: Insights into the Evolution and Taxonomic Identification of Two Weedy Species
Source: PLoS One. 2014 Nov 26;9(11):e113657. doi: 10.1371/journal.pone.0113657 (PMC4245208; doi:10.1371/journal.pone.0113657)
Supplement: Figure S1 — The first 20% (A, whole chloroplast genome sequences) and 10% (B, coding sequences of single copy genes) MCMC samples have been discarded as burn in. (PPT) [file pone.0113657.s001.ppt]

## Slide 1
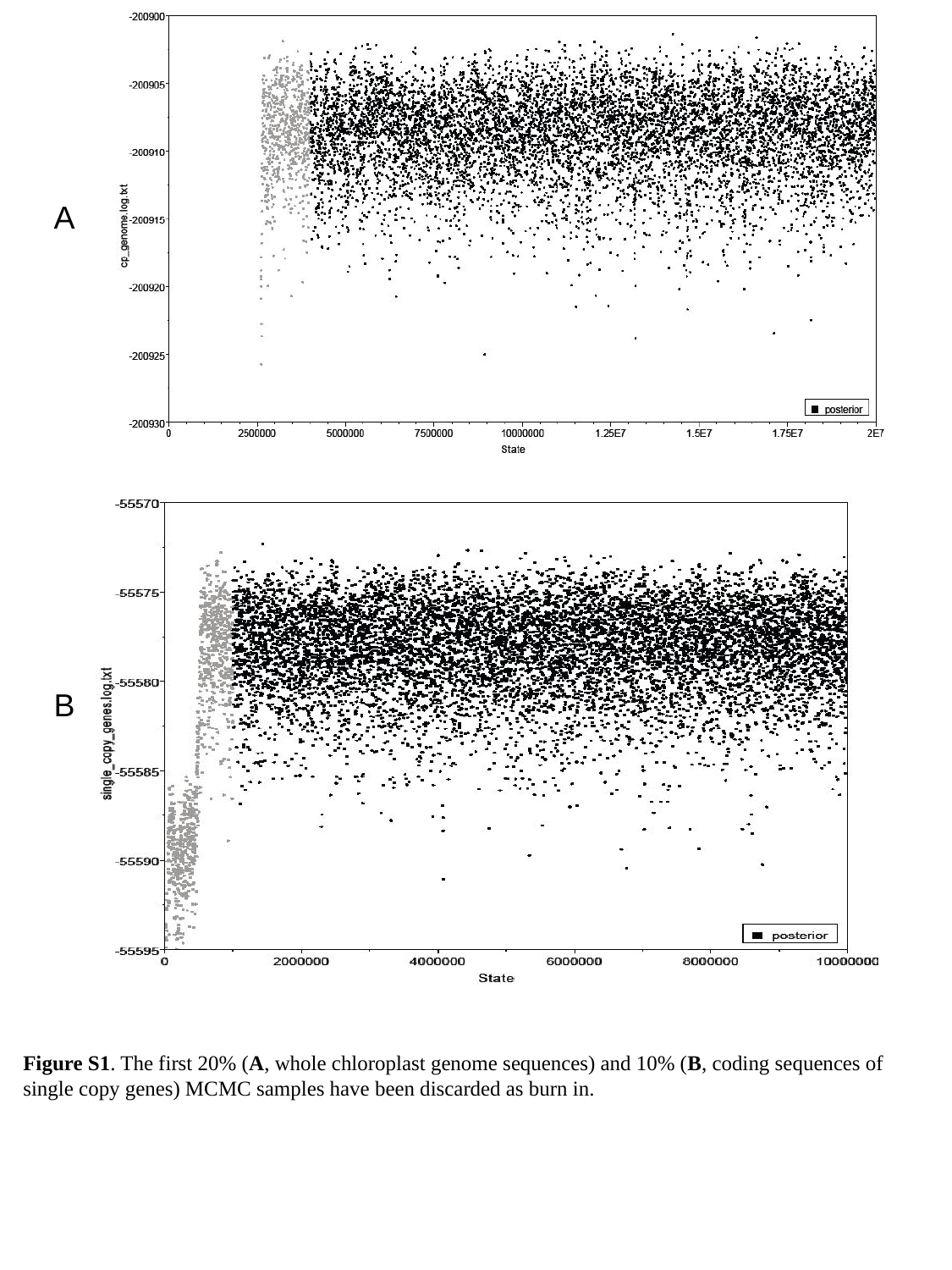

A
B
Figure S1. The first 20% (A, whole chloroplast genome sequences) and 10% (B, coding sequences of single copy genes) MCMC samples have been discarded as burn in.
