# Supplementary material for: Echinochloa Chloroplast Genomes: Insights into the Evolution and Taxonomic Identification of Two Weedy Species
Source: PLoS One. 2014 Nov 26;9(11):e113657. doi: 10.1371/journal.pone.0113657 (PMC4245208; doi:10.1371/journal.pone.0113657)
Supplement: Figure S2 — The images of STB03and BTS02 show that the former has some typical morphological traits of E. oryzicola , such as a compact plant type, bigger seeds, and high similarity with rice, whereas the latter has traits of E. crus-galli , such as geniculate culms, smaller seeds, and a loose type. (PPT) [file pone.0113657.s002.ppt]

## Slide 1
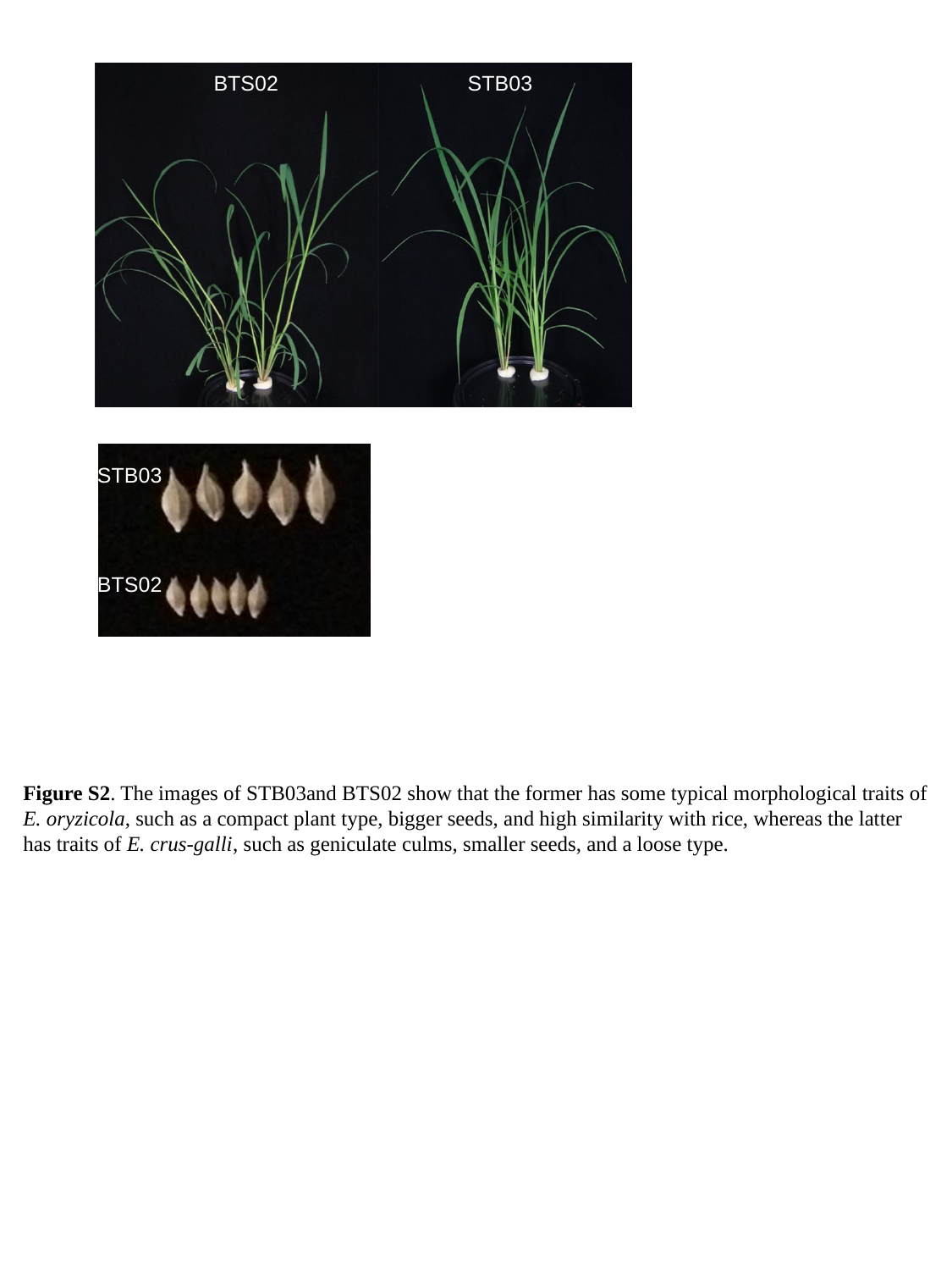

BTS02
STB03
STB03
BTS02
Figure S2. The images of STB03and BTS02 show that the former has some typical morphological traits of E. oryzicola, such as a compact plant type, bigger seeds, and high similarity with rice, whereas the latter has traits of E. crus-galli, such as geniculate culms, smaller seeds, and a loose type.
