# Supplementary material for: Echinochloa Chloroplast Genomes: Insights into the Evolution and Taxonomic Identification of Two Weedy Species
Source: PLoS One. 2014 Nov 26;9(11):e113657. doi: 10.1371/journal.pone.0113657 (PMC4245208; doi:10.1371/journal.pone.0113657)
Supplement: Figure S3 — Chromosome numbers show that STB03 (2n = 4x = 36) is tetraploid (A) and BTS02 (2n = 6x = 54) is hexaploid (B). The numbers were determined by the conventional acetocarmine method. (PPT) [file pone.0113657.s003.ppt]

## Slide 1
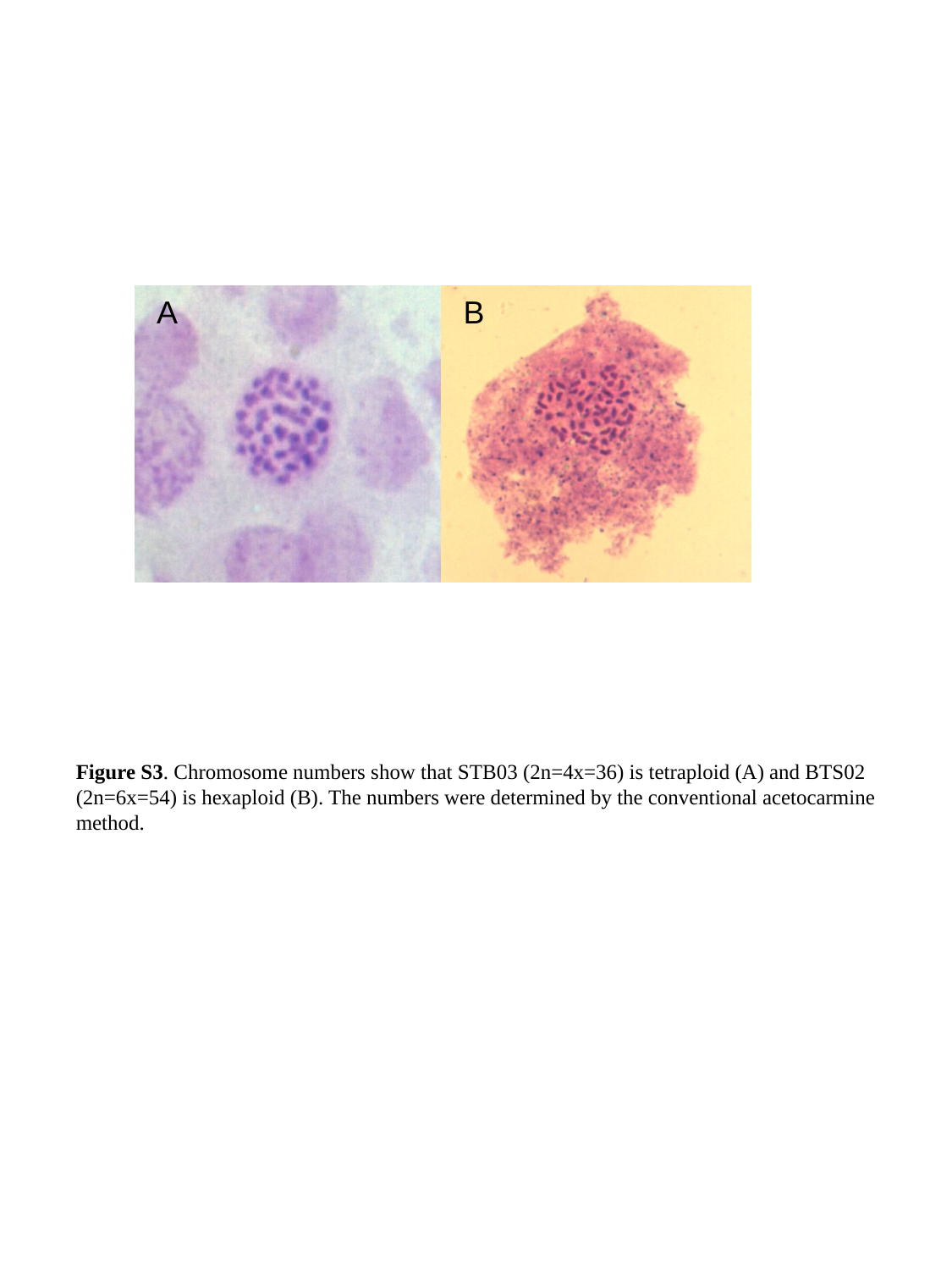

A
B
Figure S3. Chromosome numbers show that STB03 (2n=4x=36) is tetraploid (A) and BTS02 (2n=6x=54) is hexaploid (B). The numbers were determined by the conventional acetocarmine method.
