# Supplementary material for: Echinochloa Chloroplast Genomes: Insights into the Evolution and Taxonomic Identification of Two Weedy Species
Source: PLoS One. 2014 Nov 26;9(11):e113657. doi: 10.1371/journal.pone.0113657 (PMC4245208; doi:10.1371/journal.pone.0113657)
Supplement: Figure S4 — Phylogenetic relationships among STB03, BTS02, and 30 Echinochloa accessions based on the nucleotide sequences of the trn T-L-F region of the chloroplast genome. The tree was constructed using the ML method. Bootstrap values with less than 50 are not shown. (PPT) [file pone.0113657.s004.ppt]

## Slide 1
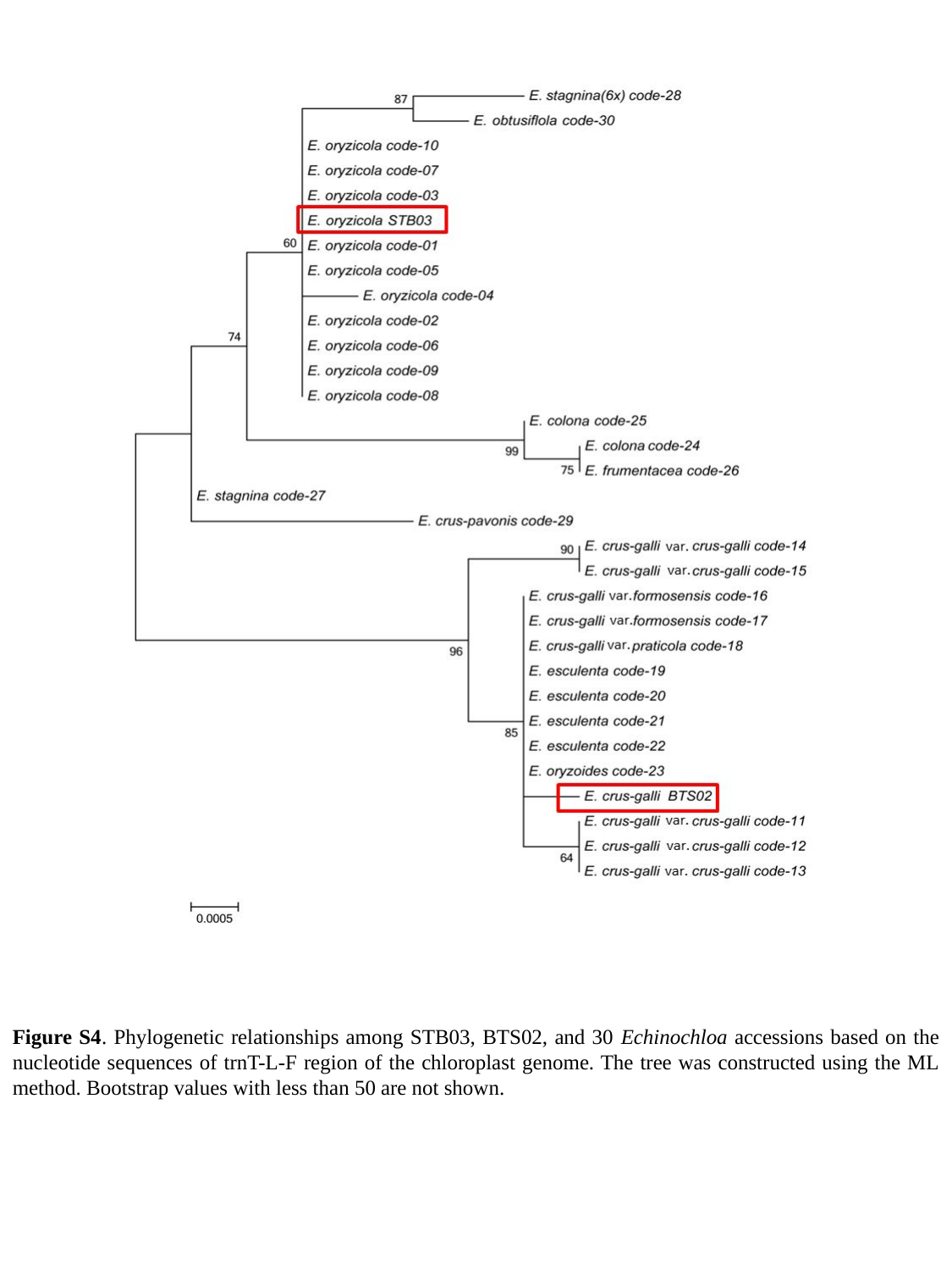

Figure S4. Phylogenetic relationships among STB03, BTS02, and 30 Echinochloa accessions based on the nucleotide sequences of trnT-L-F region of the chloroplast genome. The tree was constructed using the ML method. Bootstrap values with less than 50 are not shown.
