# Supplementary material for: Echinochloa Chloroplast Genomes: Insights into the Evolution and Taxonomic Identification of Two Weedy Species
Source: PLoS One. 2014 Nov 26;9(11):e113657. doi: 10.1371/journal.pone.0113657 (PMC4245208; doi:10.1371/journal.pone.0113657)
Supplement: Figure S5 — The E. crus-galli chloroplast genome structure and annotation. Outer circle: The genes shown outside of the circle are transcribed clockwise, whereas those inside are transcribed counterclockwise; Inner circle: the genomic structure with two inverted repeats (IR) and two single copy regions (LSC and SSC). Genes belonging to different functional groups are color coded. (PPT) [file pone.0113657.s005.ppt]

## Slide 1
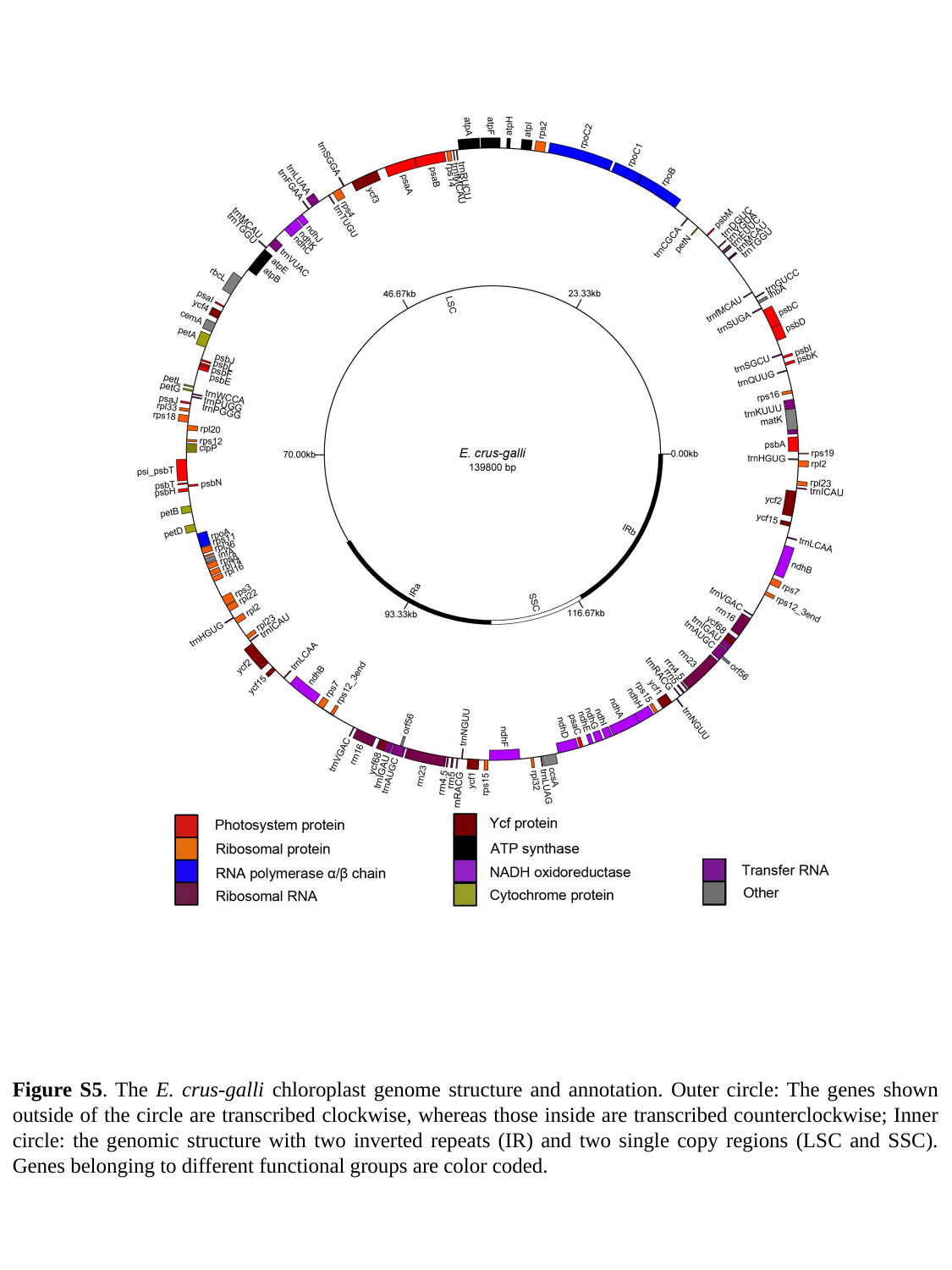

Figure S5. The E. crus-galli chloroplast genome structure and annotation. Outer circle: The genes shown outside of the circle are transcribed clockwise, whereas those inside are transcribed counterclockwise; Inner circle: the genomic structure with two inverted repeats (IR) and two single copy regions (LSC and SSC). Genes belonging to different functional groups are color coded.
