# Supplementary material for: Echinochloa Chloroplast Genomes: Insights into the Evolution and Taxonomic Identification of Two Weedy Species
Source: PLoS One. 2014 Nov 26;9(11):e113657. doi: 10.1371/journal.pone.0113657 (PMC4245208; doi:10.1371/journal.pone.0113657)
Supplement: Figure S6 — Divergence time of the genus Echinochloa . Divergence time was estimated using BEAST based on coding sequences of single copy genes shared among the six species (E. oryzicola, E. crus-galli, P. virgatum, S. bicolor, Z. mays, and O. sativa). The numbers showed at nodes indicate divergence time. (PPT) [file pone.0113657.s006.ppt]

## Slide 1
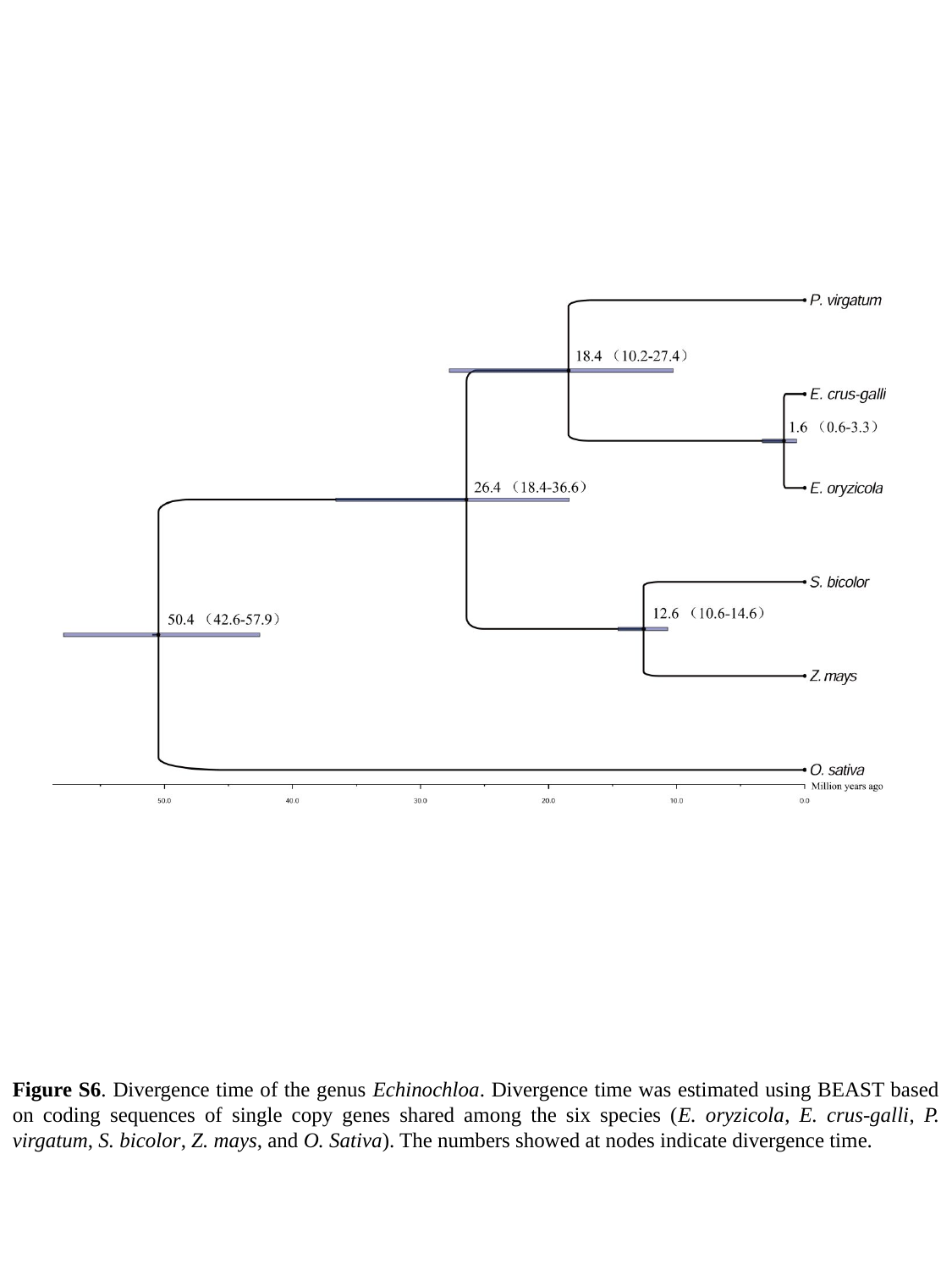

Figure S6. Divergence time of the genus Echinochloa. Divergence time was estimated using BEAST based on coding sequences of single copy genes shared among the six species (E. oryzicola, E. crus-galli, P. virgatum, S. bicolor, Z. mays, and O. Sativa). The numbers showed at nodes indicate divergence time.
